# Analyzing the regulation of metabolic pathways in human breast cancer

**DOI:** 10.1186/1755-8794-3-39

**Published:** 2010-09-10

**Authors:** Gunnar Schramm, Eva-Maria Surmann, Stefan Wiesberg, Marcus Oswald, Gerhard Reinelt, Roland Eils, Rainer König

**Affiliations:** 1 Department of Bioinformatics and Functional Genomics, Institute of Pharmacy and Molecular Biotechnology, and Bioquant, University of Heidelberg, INF 267, 69120 Heidelberg, Germany; 2 Interdisciplinary Center for Scientific Computing, University of Heidelberg, 69120 Heidelberg, Germany; 3 Theoretical Bioinformatics, German Cancer Research Center, INF 580, 69121 Heidelberg, Germany

## Abstract

**Background:**

Tumor therapy mainly attacks the metabolism to interfere the tumor's anabolism and signaling of proliferative second messengers. However, the metabolic demands of different cancers are very heterogeneous and depend on their origin of tissue, age, gender and other clinical parameters. We investigated tumor specific regulation in the metabolism of breast cancer.

**Methods:**

For this, we mapped gene expression data from microarrays onto the corresponding enzymes and their metabolic reaction network. We used Haar Wavelet transforms on optimally arranged grid representations of metabolic pathways as a pattern recognition method to detect orchestrated regulation of neighboring enzymes in the network. Significant combined expression patterns were used to select metabolic pathways showing shifted regulation of the aggressive tumors.

**Results:**

Besides up-regulation for energy production and nucleotide anabolism, we found an interesting cellular switch in the interplay of biosynthesis of steroids and bile acids. The biosynthesis of steroids was up-regulated for estrogen synthesis which is needed for proliferative signaling in breast cancer. In turn, the decomposition of steroid precursors was blocked by down-regulation of the bile acid pathway.

**Conclusion:**

We applied an intelligent pattern recognition method for analyzing the regulation of metabolism and elucidated substantial regulation of human breast cancer at the interplay of cholesterol biosynthesis and bile acid metabolism pointing to specific breast cancer treatment.

## Background

Breast cancer is a prevalent disease and a leading cause of cancer death in women [1]. Worldwide, breast cancer is the second most common type of cancer after lung cancer and the fifth most common cause of cancer death. Breast cancer patients with the same stage of disease can have very different treatment responses and overall outcome. Clinical predictive factors like age, tumor size, lymph node status, histological and pathological grade or hormone-receptor status, often fail to accurately predict clinical outcome, distant metastasis and recurrence of the cancer.

Chemotherapy and hormonal therapy reduces the risk of distant metastases by approximately one third. However, 70-80% of the patients would have survived without it (five years of follow-up) [2-4]. A more accurate means of prognosis and selection of therapy would substantially improve disease-free and overall survival of breast cancer patients [5]. Cancer cells acquire their hallmarks of malignancy through the accumulation of advantageous gene activation and inactivation events over long periods of time [6]. Nevertheless, the molecular basis of breast cancer tumorigenesis remains poorly understood. A long-standing strategy for cancer treatment is to attack basic tumor metabolism by inhibiting nucleotide biosynthesis [7,8] and DNA production [9].

Besides this, over the past decade there have been exciting developments in analyzing large scale gene expression profiles. This improved the understanding of the tumors' composition and behavior to develop new targets for therapy [1]. Many studies of gene expression identified expression profiles that are prognostic for patients with breast cancer. However, comparisons of the lists of genes derived from these studies showed that they overlap only slightly due to differences in the patient cohorts, microarray platforms, and mathematical methods of analysis [6]. One strategy to tackle this problem is to map lists of differentially expressed genes on groups of genes with related functions according to the information provided by several databases such as Gene Ontology [10] and KEGG [11]. Finding enrichments of specific gene sets related to certain phenotypes or cell states yields a functional grouping of differentially expressed genes which can be related to their pathogenic behavior and can lead to more robust results in comparison to the analysis of single genes.

To detect the enrichment of gene sets, commonly, a list of significantly differentially expressed genes is identified and statistical tests applied, such as Fisher's exact test and χ^2 ^test. In a different approach, a gene-specific statistics, known as the "local" statistics, measures the strength of association between the gene expression and the phenotype for each gene. A global statistics for a gene set is then constructed as a function of local statistics for each gene in it. The significance is assessed by permutation tests [12]. A global test for gene sets to associate gene expression with clinical outcome was presented by Goeman and co-workers and enabled determining whether the global expression pattern of a group of genes is significantly related to clinical outcome of interest using a linear regression approach [13]. In another approach, expression levels of all genes in the gene sets are combined and presented as gene specific features. These features are then compared between the treatment and the control groups to identify significantly affected gene sets [14]. In general, these methods test the association of all genes in a gene set with the phenotypes, whereas often only genes in a subset of the gene set are associated with the phenotype. Some of the genes may not belong to the set due to incompleteness or errors in the available data. Additionally, even if all genes in the gene set have apparently the same function, or belong to the same process, it is likely that only a few genes are associated with the phenotype.

To overcome these and other gene-associated problems, transcriptional data was analyzed using topology information of cellular networks. Topological information derived from the metabolic network was connected by calculating Z-scores of highly correlated sub-networks [15]. Chuang and co-workers improved classification of breast cancers with expression patterns of small subnets of a signal transduction network [16]. Substantial new genetic mediators for prostate cancer were found using reverse engineered gene networks in combination with gene expression profiles [17]. Rapaport and co-workers found gene expression patterns of neighboring genes in the network yielding good classification of the profiled samples by calculating Fourier transformations and rejecting high frequency signals [18]. Common gene expression levels of neighboring nodes in a metabolic network were calculated by averaging over all neighbors of a gene, and revealed several interesting regulated pathways for the human immune system [19]. However, these approaches were not developed to detect highly contrasting expression of neighboring genes that undergo a switch-like shift of regulation in a tumor cell. Importantly, especially these switches can be highly relevant to identify potential drug targets that specifically attack the tumor at nodes at which it redirects fluxes in the network to establish parasitic advantages.

In our study, gene expression profiles of breast tumors having an "unfavorable" prognosis were compared to breast tumors with a "favorable" prognosis. We wanted to track how the aggressive (unfavorable) tumors have specifically regulated their metabolism to optimize their oncogenetic fitness, and to elucidate ways to severely perturb this process. For this, we used an approach that detects orchestrated regulation of neighboring enzymes in the metabolic network. We mapped gene expression data onto optimally arranged grid representations of pathways of the metabolic network and applied Haar wavelet transforms onto defined pathways of the network to combine gene expression values from neighboring enzymes. These combined features were tested using a non-parametric test (Wilcoxon) if they could separate samples from different treatments. Metabolic pathways were selected that had features with the most discriminative gene expression patterns. We detected a substantially higher number of significant gene expression patterns in comparison to commonly used enrichment tests. We revealed 19 significant metabolic pathways including increased purine and pyrimidine biosynthesis which were needed for increased mitosis cycles. Furthermore, we found pathways for increased energy metabolism (glycolysis, pyruvate metabolism and fructose/mannose metabolism). Interestingly, we observed the regulation of a possible a cellular switch in the pathway for bile acid biosynthesis redirecting the metabolic flux to the synthesis of steroids while preventing degradation into bile acids.

## Results and Discussion

**Figure 1:**
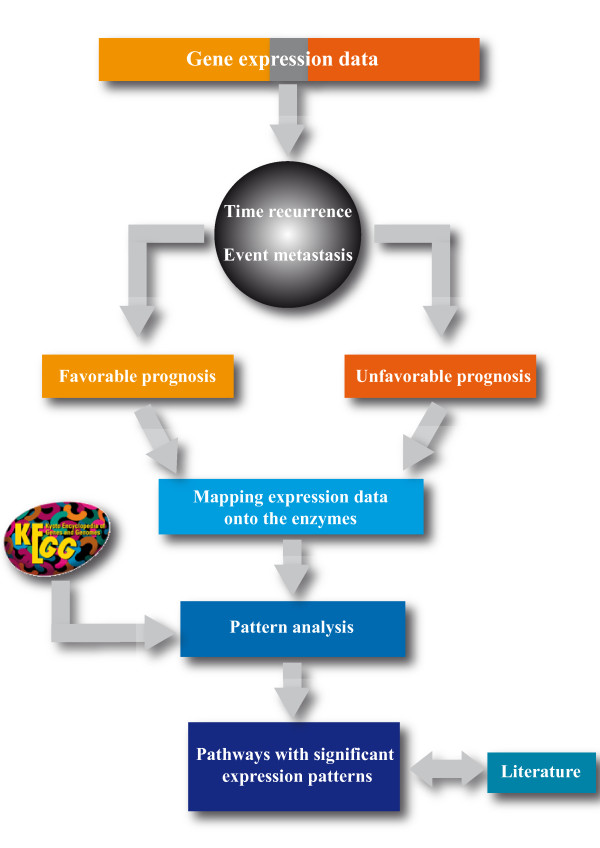
**The workflow**. Samples were divided into patients with favorable and unfavorable prognosis according to their time of recurrence (time period of relapse after the first event of breast cancer, denoted in years) and event of metastasis (favorable: time recurrence above 5 years, unfavorable: time recurrence of less than three years and the occurrence of metastasis). Expression data were mapped onto the reactions from KEGG and analyzed. Pathways with significant expression patterns were ranked according to the significance of the patterns and compared with the literature.

**Figure 2:**
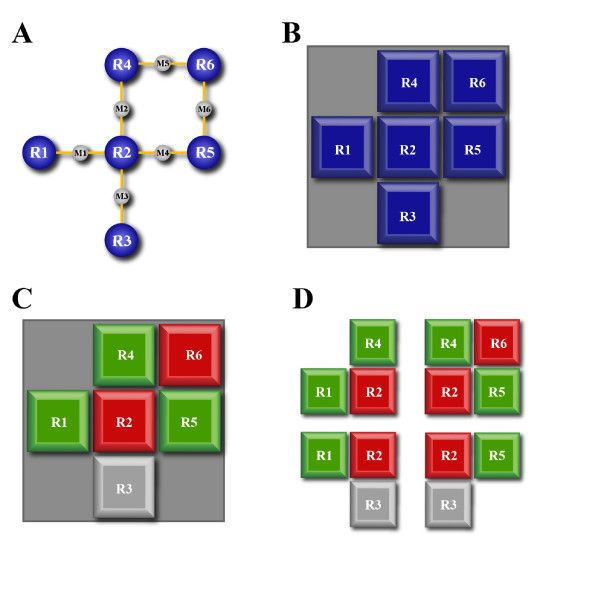
**Compiling the features**. A. We sketch the method with a simple example pathway consisting of six reactions R1-R6 and six metabolites M1-M6. B. To apply the pattern analysis method (wavelet transforms), the pathway needed to be represented on a two dimensional lattice grid. Reactions were optimally arranged to preserve next nearest neighborhoods while minimizing the distances of neighboring reactions. Metabolites didn't need to be displayed in this representation but rather used to determine the neighborhoods (e.g. R1 is a neighbor of R2 because it produces M1 which is needed as a substrate for R2 or *vice versa*). C. Gene expression data was mapped onto the corresponding enzymatic reactions. In this example genes of enzymes for reactions R1, R4, R5 were high expressed and of enzymes for reactions R2 and R6 low expressed. D. Combined gene expression features were assembled by Haar wavelet transforms which basically calculated additive and subtractive combinations of 2 × 2 pixels of the grid (pixels without reactions were filled with zeros). The figure shows all four possible arrangements of 2 × 2 pixels for which the wavelet transforms were calculated. The same procedure was done for all tumor samples. The feature which best separated the tumor entities (favorable from unfavorable) was selected for the significance of this pathway.

In this study, 250 patients were examined, 196 having a "favorable" and 54 patients an "unfavorable" prognosis. 1826 reactions could be extracted from KEGG [11] for 1771 out of which expression values could be assigned. The workflow of the method is depicted in Figure 1. Pathway maps from KEGG were represented as two-dimensional lattice grids with densely packed reactions. The reactions were arranged in a way that their neighborhoods in the network were preserved as optimally as possible (using the grid arrangement method, for details see methods). Gene expression data was mapped onto the reactions representing the according expressed enzymes. Wavelet transforms were used to combine expression values of neighboring reactions by all possible combinations of subtractions and additions. The out-coming features were tested (Wilcoxon rank test) for their possibility to discriminate between the two tumor entities (favorable and unfavorable). Figure 2 illustrates the principle of the pattern analysis for an example pathway. Pathways with the best discriminating features were selected. We revealed significant features from 19 different pathways (Table 1), including pyrimidine, purine, aminoacyl-tRNA metabolism, pyruvate metabolism, glycolysis/gluconeogenesis and fructose/mannose metabolism. These pathways have been expected as they accounted for higher biosynthesis of nucleic acids and proteins and higher energy demands of the aggressive (unfavorable) tumors. We also revealed less expected differentially regulated pathways, such as biosynthesis of steroids and bile acids. We compared the performance of our algorithm with commonly used enrichment methods. Fisher's exact tests revealed only one pathway (Lysine biosynthesis, P = 0.048) to be significantly enriched with differentially regulated genes (P ≤ 0.05, threshold for defining the differentially expressed genes also P = 0.05). In addition, we applied the well established Gene Set Enrichment Analysis (GSEA [20], two sided) to the data yielding also only one significantly enriched pathway, i.e. the pyrimidine metabolism (P = 2.2E - 03). We compared the performance of our method to Fisher's exact tests and GSEA with simulated data yielding considerably higher sensitivity for our method (see Additional file 1 A2 and Receiver Operator Characteristics: Figure A2). In the following, we will discuss the oncogenetic relevance of the pathways we found.

**Table 1:** Significantly differentially regulated pathways.

Rank	Pathway	Differentially regulated	Number of reactions	Down-regulated in unfavorable	Up-regulated in unfavorable	P-value
**1**	**Histidine metabolism**	**6**	**14**	**3**	**3**	**1.15E-05**
2	Alanine and aspartate metabolism	8	19	2	6	1.56E-05
**3**	**Valine, leucine and isoleucine degradation**	**13**	**36**	**10**	**3**	**3.74E-05**
4	Pyrimidine metabolism	37	75	3	34	2.25E-04
5	Fatty acid metabolism	14	36	9	5	2.48E-04
6	Biosynthesis of Steroids	10	41	2	8	3.12E-04
7	Methionine metaboplism	4	12	2	2	7.36E-04
8	Purine metabolism	17	91	3	14	1.21E-03
9	Glycine, Serine and Threonine metabolism	12	31	4	8	1.28E-03
10	Propanoate metabolism	4	18	3	1	2.12E-03
11	Lysine Biosynthesis	6	6	5	1	2.33E-03
12	Aminoacyl-tRNA biosynthesis	5	20	1	4	2.83E-03
13	Glycolysis/Gluconeogenesis	17	32	1	16	3.85E-03
**14**	**Bile acid biosynthesis**	**10**	**28**	**4**	**6**	**5.26E-03**
15	Pentose phosphate pathway	5	20	1	4	8.84E-03
16	Inositol phosphate metabolism	8	25	1	7	9.75E-03
17	Pyruvate metabolism	6	27	2	4	1.20E-02
18	Fructose and mannose metabolism	11	23	1	10	1.55E-02
**19**	**Galactose metabolism**	**8**	**24**	**4**	**4**	**2.00E-02**

Pathways with significant regulation patterns and more than three differentially regulated reactions (as defined by KEGG), bold: pathways which had also significant patterns in the second analyzed dataset.

### Pyrimidine and purine metabolism

Most up-regulated reactions were identified in the pyrimidine (P = 2.25E - 04) and purine (P = 1.21E - 03) metabolism. These pathways were up-regulated to enable enforced nucleotide biosynthesis for increased cell cycle activity of the aggressive tumors. Nearly all enzymes involved in the biosynthetic pathway for nucleotides were up-regulated, such as enzymes converting substrates to dNTPs and polyribonucleotide nucleotidyltransferases (EC 2.7.7.7), incorporating dNTPs into DNA. Enzymes reversing pyrimidine and purine anabolism, such as enzymes degrading dNTPs, were down-regulated (ECs 3.6.1.17, 1.3.1.2 and 2.7.4.3). Reactions involved in RNA synthesis were partially up-regulated to increase protein biosynthesis (EC 2.7.7.8). Furthermore, reactions which were responsible for synthesizing adenosine were up-regulated (EC 2.4.2.1). Adenosine was shown to be angiogenic, cyto-protective and anti-inflammatory in several tissues and contributed to more aggressive behavior and metastasis of cancer cells [21].

### Pathways for energy supply were significantly up-regulated

We detected significant differential expression patterns in glycolysis (P = 3.85E - 03), pyruvate (P = 1.20E - 02) and fructose/mannose (P = 1.55E - 02) metabolism. They were mostly up-regulated to generate sufficient energy and metabolites for fast-growing cancer cells. Genes of the glycolysis pathway have been found to be over-expressed in a set of 24 cancers, while other pathways showed less consistent up-regulation so far [22]. Glycolysis is increased in cancers and this generates ATP by oxidative phosphorylation [23,24]. Furthermore, elevated glycolytic flux may support the production of important metabolites that contribute to essential cellular processes like fatty acid synthesis, nucleotide synthesis and to the better protection of oxidative stress [25,26].

However, the extent of up-regulated genes in glycolysis varies for different cancer types. In our study, all except one differentially regulated reactions were up-regulated, including enzymes such as glyceraldehyde-3-phosphate dehydrogenase (EC 1.2.1.12), phosphor glycerate mutase (EC 5.4.2.1), phosphoglycerate kinase (EC 2.7.2.3), triosephosphate isomerase (EC 5.3.1.1) and fructose-bisphosphate aldolase (EC 4.1.2.13). These enzymes were also described as up-regulated by Altenberg and co-workers [22]. Inhibitors of glycolysis, such as the glucose analog 2-deoxyglucose, which binds and suppresses hexokinase I, and arsenate, that causes arsenolysis in glyceraldehyde-3-phosphate dehydrogenase, as well as 3-bromopyruvate, an inhibitor of hexokinase II, have already been developed to target this metabolic abnormality and could effectively kill cancer cells [27].

Inhibition of glycolysis was effective even in killing cancer cells with a multidrug resistance (MDR) phenotype. It is known that cells expressing MDR proteins require ATP as their energy source to export drugs out of the cell. Thus, to overcome this drug resistance, depletion of cellular ATP causes the excretion to fail and consequently, cancer cells become more sensitive to anti-cancer therapy [27].

We found a smaller pattern of up-regulated reactions in the pyruvate metabolism. Significantly up-regulated were: pyruvate kinase (EC 2.7.1.40) which is responsible for ATP production in glycolysis, and the conversion of pyruvate to malate via (S)-Malate:NADP+ oxidoreductase (ECs 1.1.1.38, 1.1.1.39, 1.1.1.40). Aldehyde dehydrogenase (EC 1.2.1.3) was down-regulated to prevent degradation of fatty acids. Oxaloacetate was reported to directly induce cell proliferation by increasing DNA synthesis [28]. Surprisingly, our results did not show any increase in lactate dehydrogenase, which is often up-regulated in cancer cells [22]. Highly expressed reactions in fructose and mannose metabolism were mainly involved in the conversion of D-fructose (EC 4.1.2.13), D-fructose-1P (ECs 2.7.1.1, 2.7.1.11, 4.1.2.13) and D-mannose (ECs 2.7.1.1, 2.7.1.11, 4.1.2.13) into glyceraldehyd-3P, a substrate of glycolysis, leading to increased ATP production.

### Biosynthesis of steroids and bile acids

**Figure 3:**
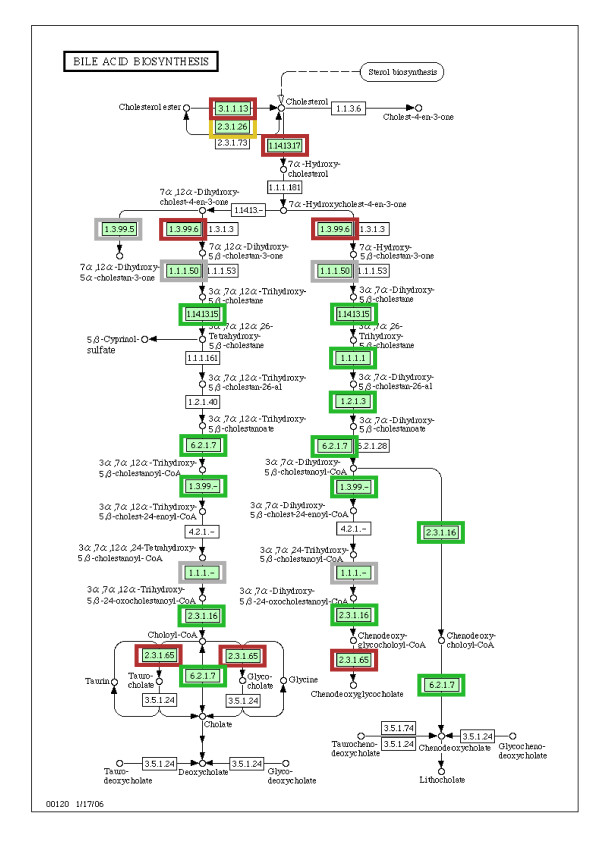
**Regulation of the pathway for bile acid biosynthesis of both analyzed datasets**. Red frame indicates up-regulation in at least one dataset, green frame down-regulation in at least one dataset, yellow frame up-regulation in the two respective datasets, and grey frame down-regulation in the two datasets, respectively, and grey frame no differential regulation in both datasets. The map was taken from KEGG [11]. The lower part of this pathway was mainly down-regulated to prevent degradation of steroids into bile acids, whereas the upper part was mainly up-regulated to support steroid metabolism.

The steroid pathway was mainly up-regulated in breast cancer of unfavorable outcome. 8 out of 10 reactions were significantly up-regulated (8 reactions from KEGG comprising ECs 2.5.1.29, 2.5.1.10, 2.5.1.1, 1.1.1.170, 5.3.3.5, 1.3.1.21). Cholesterol is a precursor for the biosynthesis of steroid hormones. It was shown that sex steroid hormones such as estrogen increase the proliferation of breast cancer cells by acting on estrogen receptors [29]. Directly connected with the production of cholesterol is the biosynthesis of bile acids. Figure 3 shows the regulation of bile acid biosynthesis (including the results from the second analyzed dataset, the second analyzed dataset is described in the next section). In the bile acid biosynthesis pathway, reactions generating cholesterol and its derivates were up-regulated (ECs 3.1.1.13 and 2.3.1.26), whereas the lower part of the pathway was mainly down-regulated, i.e. the production of bile acids such as cholate and lithocholate (ECs 2.3.1.16, 1.2.1.3). Conversion of cholesterol to bile acids is the major pathway for cholesterol catabolism in the human body [30]. Therefore, down-regulation of the lower part of the bile acid pathway and up-regulation of cholesterol biosynthesis may, in conjunction, support steroid biosynthesis which supports estrogen mediated tumorigenesis of breast cancer cells.

Besides this, there have been intensive cancer investigations on bile acids as they have very heterogeneous effects on tumor cells and carcinogenesis. They are known enhancers for invasiveness of colon cancers [31,32]. In turn, bile acids can induce apoptosis either specifically (receptor-mediated interactions) [33] or, in high concentrations, non-specifically (as detergents) through mitochondrial destabilization and oxidative stress [34]. Non-toxic doses of deoxycholic acid (DCA), chenodeoxycholic acid (CDCA) and lithocholic acid (LCA) induced differentiation in promyelocytic leukemia cell lines [35]. 6ECDCA is a synthetic bile acid derivative and can act as a selective FXR ligand to promote differentiation of preadipocyte cell lines [35]. In the blood plasma of postmenopausal women with newly diagnosed breast cancer, elevated concentrations of DCA were detected which may have been released from osteoblasts to induce migration of the cancer [30]. This would explain a down-regulation in breast cancer cells as they may use elevated bile acid levels from the blood for detaching and infiltrating while sustaining their endogenous cholesterol synthesis.

Bile acids are normally predominantly produced by hepatocytes which may explain the elevated levels of bile acids in the blood when elevated breast cancer estrogen is decomposed into bile acids in the liver. In conclusion, this regulation may reflect a cellular switch which redirects the metabolic flux from degradation of cholesterol (via bile acids) into the conversion of steroid hormones, suggesting drug targets in the biosynthesis of steroids, such as mevalonate (diphospho) decarboxylase (EC 4.1.1.33) and mevalonate kinase (EC 2.7.1.36).

### Analyzing a second dataset

To verify our findings we analyzed an independent gene expression dataset of breast cancer [36,37] consisting of 414 samples. The data was downloaded from Array Express (GSE6532, http://www.ebi.ac.uk/microarray-as/ae/) and normalized as described in Methods. We divided the dataset into 250 tumors showing a favorable prognosis and 61 tumors with an unfavorable prognosis following our criteria as described for the other dataset (in Methods). 103 tumors were discarded as they did not fit in any of the prognostic groups. The significance level was again set to P = 0.05. Our analysis revealed 12 pathways with significantly differentially regulated patterns (see Additional file 1 Table A1). Although the overlap of survival predicting genes between different breast cancer studies is often rather small [38], four pathways from the first analyzed study also showed a significant regulation pattern in this second dataset, i.e. histidine metabolism, valine, leucine and isoleucine degradation, bile acid biosynthesis and galactose metabolism. Interestingly also the citrate cycle showed a significant regulation pattern which compares to the pathways for carbon hydrate metabolism we found in the first analyzed dataset, and C_21_-steroid hormone metabolism which compares to steroid biosynthesis found in the first analyzed study.

## Conclusion

Performing a network based gene expression analysis revealed interesting insights into the metabolism and regulation of aggressive breast tumor cells. Expected differentially regulated pathways in cancer could be confirmed, e.g. the aggressive tumors showed significantly up-regulated pathways for purine and pyrimidine synthesis to maintain elevated proliferation, as well as the up-regulation of glycolysis and pyruvate metabolism to support energy supply for the tumor. The analysis revealed insights into differentially regulated metabolic pathways in breast cancer cells, which may support the induction of proliferation by inositol signal transduction cascades (inositol phosphate metabolism) and steroid hormones (biosynthesis of steroids). An interesting view in breast cancer metabolism was observed in the interplay of biosynthesis of steroids and bile acids, the latter of which was down-regulated possibly to convert cholesterol into proliferative acting steroid hormones. Such a cellular switch would be interesting to compare to other tumors and tissues, also in order to define a tumor specific therapy. Performing Fisher's exact tests as a standard enrichment analysis revealed the lysine biosynthesis pathway to be significantly enriched with differentially expressed genes. The GSEA method yielded pyrimidine biosynthesis. In addition to this pathway, our method yielded eighteen further significant regulation patterns. However, not all pathways detected by such enrichment tests and also our pattern analysis may be relevant for the pathogenesis of the analyzed disease. Some pathways in our results, such as valine, leucine and isoleucine degradation, could not be associated with oncogenesis and may need further examination.

Complex regulated pathways which were relevant to breast tumors with unfavorable prognosis were detected and described in a straightforward manner. The global analysis of network patterns offered a good insight into the regulation of metabolism in breast tumors and may support revealing new potential targets for drug design, specifically in the interplay of the biosynthesis of bile acids and steroids.

## Methods

We wanted to identify pathways and regions of pathways that showed patterns of differentially regulated reactions. To find such patterns we applied wavelet transforms on the mapped gene expression data. For the application of wavelet transforms and the subsequent feature calculations we needed to arrange the metabolic pathways onto a 2-dimensional grid (Figure 2 illustrates this procedure with a small example pathway).

### Preparing the microarray data

Normalized gene expression data was taken from a published study [5] of breast-cancer samples of 295 women (diagnosis between 1984 and 1995) with age ≤ 53 years and no previous history of cancer, except for non-melanoma skin cancer. The data was downloaded from Rosetta Inpharmatics at http://www.rii.com/publications/2002/nejm.html. The gene expression profiles were derived by using oligonucleotide microarrays from Agilent Technologies http://www.agilent.de. Data on relapse-free survival (defined as the time to the first event) and overall survival were available for all patients. Most of the patients had breast cancer of stages one and two. 165 had received local therapy alone, 20 had received tamoxifen only, 20 had received tamoxifen plus chemotherapy, and 90 had received chemotherapy only. 151 patients were diagnosed as lymph-node-negative and 144 as lymph-node-positive. The tumors were primary invasive breast carcinoma that were less than 5 cm in diameter at pathological examination [5]. To differentiate between tumors with "favorable" and "unfavorable" prognosis, samples were separated according to their "time recurrence" (period of time of relapse after the first event of breast cancer, denoted in years) and "event of metastasis" (if metastasis occurs during this period). A time recurrence above 5 years indicated a "favorable" prognosis (196 patients), whereas samples were classified as samples with unfavorable prognosis if they showed a time recurrence of less than three years and the occurrence of metastasis (54 patients). Samples with ambiguous information were discarded (45 samples) and identified either by a time recurrence of less than three years without the event of metastasis or by a time recurrence above three years but below five years. A Remark: A new ethical approval was not needed for our study as all our analyses based on published microarray datasets (which also applies to the second analyzed dataset).

### Assembling the metabolic pathways

Pathways were defined according to curated pathway maps of the KEGG database [11] (version from February 4^th^, 2009). Two reactions were neighbors if a metabolite existed that was the product of one and the substrate of the other. We defined reactions as the nodes and metabolites as the edges between them. Pathways without any connected reaction were discarded. This resulted in 99 pathways with 1826 different reactions. Each pathway was represented by its adjacency-matrix. An entry at row *a *and column *b *was set to one if there existed a metabolite that was produced by reaction *a *and consumed by reaction *b *or vice versa. The sizes of the symmetric adjacency-matrices were between 2 × 2 and 92 × 92 reactions.

### Ordering the two-dimensional pathway representation with the grid arrangement method

To apply our feature extraction method we required a 2-dimensional grid arrangement of the metabolic network. We calculated an embedding of the metabolic networks for every KEGG pathway into a 2-dimensional, regular square lattice grid. To preserve neighborhood characteristics of the reactions, we were looking for embeddings in which adjacent nodes of the network were placed onto the grid as close to each other as possible. We wanted to determine an optimal neighborhood in which the total edge length of the graph was minimized according to some metric on the lattice. The network topology was preserved as good as possible. For this purpose the minimization of total edge length is more suitable than a minimization of the longest edge which is widely applied in very large scale integration (VLSI) designs as the latter one allows a variety of optimal solutions in which adjacent nodes are placed unnecessarily far from each other. As a measure of distance in the lattice, we used the natural metric induced by the underlying lattice graph, the so-called Manhattan distance. That is, for any two grid points *u *= (*i*_1_, *j*_1_) and *v *= (*i*_2_, *j*_2_) the distance was given by *d_uv _*= |*i*_1 _- *i*_2_| + |*j*_1 _- *j*_2_|. This resulted in an NP-hard combinatorial optimization problem. We stated this problem as an integral linear program (IP) (see [39] for an introduction to integer programming). We formulated the IP by introducing 3-dimensional binary variables *x_vij _*for every node *v *and every grid point (*i, j*) stating whether or not node *v *has to be placed on grid point (*i*, *j*). For each pair of nodes (*u*, *v*) we calculated their distance *d_uv_*. For a given lattice grid *g*, the undirected network graph *G *= (*V*, *E*) with node set *V*, edge set *E *and adjacency matrix *M*, the most basic IP was given by finding an optimum for

(1) $$\underset{x,d}{\min}\sum_{a,b\in V,a<b}M(a,b)\cdot d_{ab},$$

with the constraints

(2) $$\begin{array}{cc} \sum_{(i,j)\in g}x_{vij}=1 & \text{for all }v\in V \end{array},$$

(3) $$\begin{array}{cc} \sum_{v\in V}x_{vij}\leq 1 & \text{for all }(i,j)\in g \end{array}$$

(4) $$\begin{array}{cc} d_{ab}\geq A+B, & d_{ab}\geq A-B \end{array}$$

(5) $$\begin{array}{cc} d_{ab}\geq -A+B, & d_{ab}\geq -A-B \end{array}$$

for all (*a*, *b*) ∈ *V *× *V*, *a *<*b*, where

(6) $$\begin{array}{cc} A & :=\sum_{(i,j)\in g}i\cdot x_{aij}-\sum_{(i,j)\in g}i\cdot x_{bij}, \end{array}$$

(7) $$\begin{array}{cc} B & :=\sum_{(i,j)\in g}j\cdot x_{aij}-\sum_{(i,j)\in g}j\cdot x_{bij}, \end{array}$$

(8) $$\begin{array}{ccc} x_{vij}\geq 0, & x_{vij}\in Z & \text{for all }v\in V,(i,j)\in g. \end{array}$$

Constraints Eq. (2) and Eq. (3) guaranteed that all nodes (reactions of the pathway) were placed exactly once and that each grid point could be used at most once, thus avoiding multiple placements and stacking of reactions on a single grid point. Constraints Eq. (4) and Eq. (5) ensured that the distance of node *a *and *b *is given by *|A| + |B| *where *A *and *B *are computed by Eq. (6) and Eq. (7) as *A = i_a _- i_b _*and *B = j_a _- j_b_*. All variables were enforced to values 0 or 1 by constraint Eq. (8). The problem was solved by CPLEX 8.1 (ILOG, Gentilly, France) for 99 lattice grids (representing 99 KEGG-maps) with an average optimality of 96% for embeddings on square grids of side length $\sqrt{\left|V\right|}+1$, rounded up to the next integer. By choosing a grid of the smallest possible size, we reduced both the number of variables in the model and the number of unoccupied sites on the grid. This basic model was enhanced by a number of graph dependent, additional constraints on the distance variables. They provided lower bounds for the distance sums of well-known sub-graph motifs. For an edge induced sub-graph *G*' ⊂ *G *with a least objective function contribution of *lb*(*G*'), the following inequality was added or dynamically separated by

(9) $$\sum_{\left(u,v)\in E(G'\right)}d_{uv}\geq lb(G')$$

The right-hand sides *lb*(*G*') for the different sub-graph motifs needed to be determined only once as they are independent of G. Furthermore, the motifs were defined in a pre-processing step and could therefore be separated quickly during the optimization process. We considered the sub-graph motifs of star graphs, cliques consisting of up to 10 vertices and odd cycles (2k + 1-cycles) for k = 1,2. Moreover, a certain class of trees with maximum vertex degree Δ(T) ≤ 4 decreased computation time and enhanced separation ability. Furthermore, calculation time was reduced by symmetry breaking constraints eliminating all but a few representative embeddings from each equivalence class of symmetrical embeddings. For this, grid symmetries due to translation, rotation and reflection of the embeddings were considered as well as vertex subsets which inner permutations didn't change the value of the objective function. After solving the optimization problem the values of the variables *x_vij _*allowed to construct the embedding of the metabolic pathway on a 2-dimensional grid placing reaction *v *on the grid point with coordinates (*i,j*). On the grid representations of the pathways we then applied our feature generation method.

### Pattern recognition of gene regulation on the metabolic network

Neighboring enzymes on the 2-dimensional grid representation were grouped by combining their gene expression values with wavelet transforms. These transforms yielded combined expression values ("features") of low pass filters to detect similar expression changes and high pass filters to detect contrasting regulation patterns. The wavelet calculation is described in the following. Each cluster-matrix was divided into 2 × 2 pixeled disjoint sub-sections (e.g. a cluster matrix of size 8 × 8 was divided into 16 sub-sections). Clusters with non-fitting sizes (e.g. 3 × 3, 5 × 5,) were extended with rows and columns of zeros to yield matrices that could be divided into 2 × 2 pixels sub-sections. For each sub-section, all combinations of row-wise and column-wise mean and differences, respectively, were calculated. This yielded 4 combined values for each 2 × 2 pixels sub-section: 1st: mean of the mean of the upper and mean of the lower row, 2nd: difference of the mean of the upper and the mean of the lower row, 3rd: mean of the difference of the upper and the difference of the lower row, and, 4th: difference of the difference of the upper and the difference of the lower row. All four combined values for each 2 × 2 pixels sub-section were stored and applied as features for the classifier. This was done for all sub-sections of the matrix. All 1st combined values (mean of means) were taken for a new matrix and were again grouped into 2 × 2 fractions that were combined in the same manner, yielding again 4 new features for every fraction. This procedure was repeated until no further grouping was possible. The discriminative behavior of all non-trivial features was tested using Wilcoxon rank tests. Pathways were ranked according to their best discriminating features. Features were regarded as significant if they had p-values ≤ 0.05 after correction for multiple testing using the Bonferroni method [40,41]. Only pathways with more than three significantly, differentially regulated reactions and genes were further investigated to focus on the most relevant features. Two reactions that consisted of exactly the same genes were counted as one reaction.

## Competing interests

The authors declare that they have no competing interests.

## Authors' contributions

GS and RK conceptualized and designed the method. GS and EMS analyzed the data. SW, MO and GR provided solutions for the combinatorial optimization problem. GS, EMS and RK wrote the manuscript. RE and RK revised it critically. All authors read and approved the final manuscript.

## Pre-publication history

The pre-publication history for this paper can be accessed here:

http://www.biomedcentral.com/1755-8794/3/39/prepub

## Supplementary Material

Additional file 1 **Additional results and performance assessment**. Results for the additional gene expression data set and comparison of our approach with standard enrichment tests using simulated data. Click here for file
