# Supplementary material for: Analyzing the regulation of metabolic pathways in human breast cancer
Source: BMC Med Genomics. 2010 Sep 10;3:39. doi: 10.1186/1755-8794-3-39 (PMC2945993; doi:10.1186/1755-8794-3-39)
Supplement: Additional file 1 — Additional results and performance assessment. Results for the additional gene expression data set and comparison of our approach with standard enrichment tests using simulated data. [file 1755-8794-3-39-S1.PDF]

Additional file 1

Table A1: Results of the second dataset

We analyzed expression data of a second breast cancer study consisting of 250 tumors with favorable prognosis and 61 tumors with unfavorable prognosis. The analysis was performed as described in the main text. Shown are the identified pathways with significant regulation patterns and more than three differentially regulated KEGG reactions. Red: pathways also found in the first analyzed study.

Rank	Pathway	p-value
1	Arachidonic acid metabolism	4.65E-04
2	Histidine metabolism	8.59E-04
3	Glycerolipid metabolism	2.28E-03
4	Tryptophan metabolism	3.31E-03
5	Tyrosine metabolism	6.14E-03
6	Ether lipid metabolism	8.06E-3
7	Valine, Leucine and Isoleucine degradation	1.02E-02
8	Bile acid biosynthesis	1.43E-02
9	Glycosaminoglycan degradation	1.85E-02
10	Citrate cycle (TCA cycle)	2.54E-02
11	Galactose metabolism	3.28E-02
12	C_{21}-steroid hormone metabolism	4.40E-02

A2: Assessing the performance of our approach

To estimate to which extend our results contained false positives we compared our approach to two established enrichment tests (Fisher's exact test and GSEA). As performance differences are difficult to quantify when using experimental data, we performed the comparison with simulated data. For each reaction, normally distributed random expression data (mean = 0, standard deviation $\sigma = 1$) was generated for 200 samples. The samples were split into two classes with 100 samples each. For each simulation between five and twenty KEGG pathways were randomly selected with 50% of their reactions randomly chosen to be differentially regulated. Differential regulation of the selected reactions was simulated by adding a constant Δ ($\Delta = 1\sigma$) to the expression data of the reactions of one class. On this data the enrichment tests were performed to assess their overall performance. In total the simulations were repeated 100 times. The overall performance was measured by calculating specificity, sensitivity and precision using a p-value cutoff of $P = 0.05$ for all runs. The method described in this paper performed better than Fisher's exact test and GSEA and showed a distinct lower amount of false positives. The results are given in Table A2. Furthermore, we computed the ROC-curve for the three tests on simulated data by varying the p-value cutoff between $P \leq 0$ and $P \leq 1$ (Figure A2).

Table A2: Comparison to standard enrichment tests

Comparison of our approach with Fisher's exact test and GSEA on simulated data. Shown is the overall performance of 100 simulation runs with a p-value cutoff of $P \leq 0.05$.

	**Sensitivity**	**Specificity**	**Precision**
**Fisher**	81.1	99.8	98.2
**GSEA**	75.1	98.6	89.4
**Our approach**	90.6	100.0	100.0

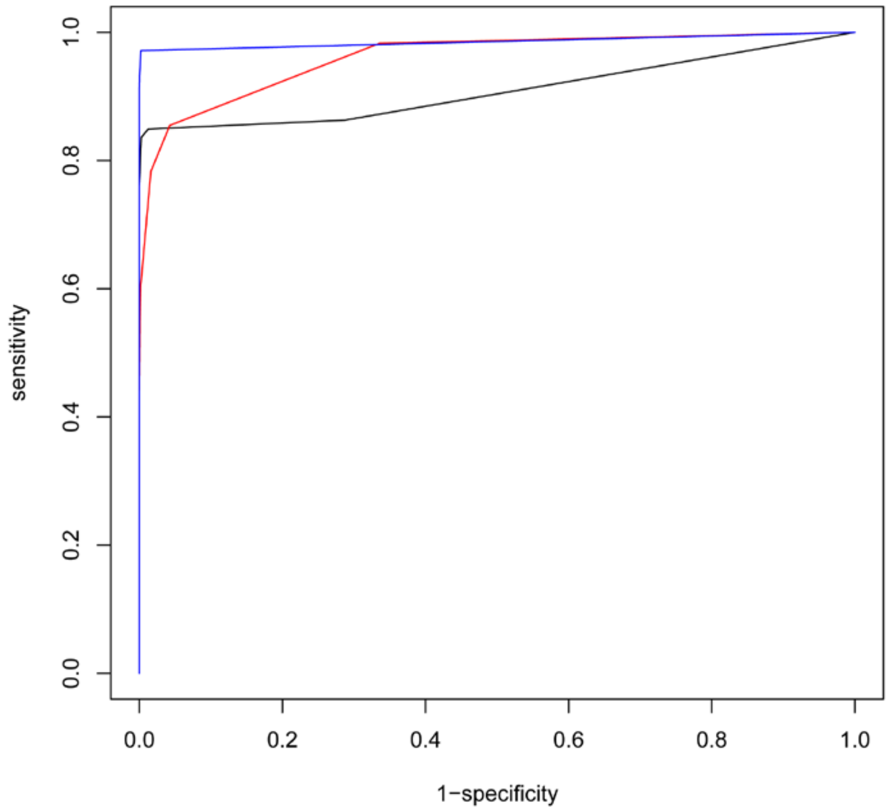

Figure A2. Receiver Operator Characteristics for simulated data. The ROC curves of three enrichment tests -Fisher's exact test (black curve), GSEA (red curve) and our approach (blue curve) were computed by varying the p-value cutoff between P≤0 and P≤1. Simulated data was generated as described above. Shown is the overall performance of 100 simulation runs for each p-value cutoff.
